# Regulatory T Cells Modulate DNA Vaccine Immunogenicity at Early Time via Functional CD4^+^ T Cells and Antigen Duration

**DOI:** 10.3389/fimmu.2015.00510

**Published:** 2015-09-29

**Authors:** Lizeng Qin, Guosheng Jiang, Jinxiang Han, Norman L. Letvin

**Affiliations:** ^1^Department of Microbiology, Shandong Academy of Medical Sciences, Jinan, China; ^2^Center for Virology and Vaccine Research, Beth Israel Deaconess Medical Center, Harvard Medical School, Boston, MA, USA

**Keywords:** regulatory T cells, immunogenicity, DNA vaccine, antigen, mice

## Abstract

The development of an effective vaccine against HIV has proved to be difficult. Many factors including natural regulatory T cells (Treg cells) can dampen the CD8 T-cell immunogenicity. In this study, we aimed to understand how Treg cells control CD8^+^ T-cell immune responses during DNA prime-boost immunization. Animals were immunized with plasmid HIV IIIB gp120 DNA following elimination of Treg cells by administration of anti-CD25 neutralizing antibody. Results demonstrated that the pool size of CD4^+^ T cells producing both IL-2 and/or IFN-γ (CD4^+^/IL-2^+^/IFN-γ^+^) was increased solely during the priming phase. An increment of tetramer binding and intracellular cytokine IFN-γ expression, however, were elevated in both primary and secondary stages in CD8^+^ T cells. The speed of antigen clearance was also investigated by using DNA luciferase. Surprisingly, DNA luciferase expression was declined to basal level over the ensuing observation period when Treg cells were depleted. Importantly, we found for the first time that DNA expression pattern in Treg-depleted animals was similar to that of the regular memory phase. Moreover, in mice that were exposed to antigen over 5 days prior to Treg cell depletion, CD8^+^ T-cell memory response was not affected. Thus, in the present study, we propose a new concept and prove that the enhanced immune response following the depletion of Treg cells during the priming phase likely adds one more set of memory response to the immune system. Taken together, our findings support the notion that Treg cells control DNA vaccine immunogenicity at an early time via antigen duration and functional CD4^+^ T-cell responses.

## Introduction

Gaining a better understanding of the mechanisms guiding the generation and differentiation of effector and memory CTL remains a top priority in vaccinology and immunology.

While enormous advances have been made in recent years in understanding how immune responses are initiated and amplified, harnessing that understanding to improve vaccines has proven difficult (1, 2). Plasmid DNA immunogens are potential candidates in the design of an effective HIV vaccine (3). However, clinical trials of DNA vaccines have shown that the immunogenicity is much lower than conventional protein- or carbohydrate-based human vaccines, suggesting that a variety of unknown factors are at work. In order to develop a vaccine with an effective CTL immune response, we have to consider several factors, such as the ability of Treg cells to suppress immune activation (4), the help of CD4^+^ T cells to initiate immune response (5–7), and the duration of antigen stimulation (2, 5–10). Since it is known that counterregulatory mechanisms can lower the capacity to mount an effective immune response, controlling these regulatory mechanisms would potentially enhance the efficacy of vaccines and the management of chronic infections (4).

Natural regulatory T cells (Foxp3^+^CD25^+^CD4^+^ T cells, Treg cells) have been implicated in counterregulatory mechanisms, and accumulating evidence suggests that controlling their growth and proliferation may improve immunogenicity. For example, a number of mechanisms that boost immune responses and favor the control of pathogens also abrogate natural Treg cell function (4). In addition, a growing body of literature demonstrates that a distinct population of Treg cells is crucial for regulating the magnitude and specificity of immunity against a large number of infections (4), including many life-threatening diseases such as AIDS, TB, hepatitis C virus infection, and malaria (11–15). Strategies that manipulate natural Treg cells function or abundance have shown therapeutic and preventive potentials (16, 17). For a number of infections, in both mice and humans, the depletion of Treg cells or molecules associated with their functions has resulted in enhanced effector immune responses (11, 13, 17). More recently, studies have also confirmed the role of Treg cells by the use of genetically engineered Foxp3-DTR mice in several laboratories to study the specific role of Treg cells in primary and memory T-cell responses following vaccination and/or infection (18, 19).

In light of previous studies, we sought to understand the role by which Treg cells restrain CD8^+^ T-cell immune responses to a specific DNA vaccine. We drastically reduced Treg cell population through the administration of anti-CD25 antibody in mice to measure their antigenic responses to plasmid luciferase and immunogenicity to the HIV DNA vaccine, respectively. Apart from the augment of immune response to the DNA vaccine, we have shown for the first time that the DNA expression pattern is similar to the one of the regular memory response when Treg cells are depleted. We conclude that Treg cells modulate CD8^+^ T-cell immunogenicity via control of antigen duration and functional CD4^+^ T-cell response during the early stage of immune response. Given the depletion of Treg cells during priming phase, the enhanced immune response is likely adding one more set of memory responses to the immune system.

## Results

### Kinetics of Tregs cells following administration of anti-CD25 monoclonal antibody

**Figure 1:**
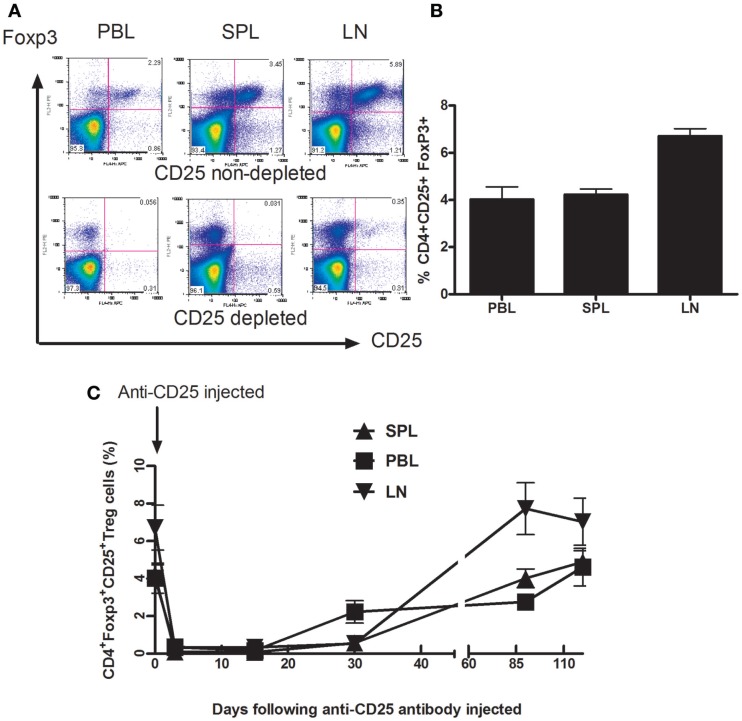
**Depletion of Foxp3^+^CD4^+^CD25^+^ T cells from Balb/C mice after *in vivo* treatment with PC61 anti-CD25 mAb**. Mice (*n* = 6 mice/group) were given a dose of 1 mg of PC 61 anti-CD25 monoclonal antibody i.p., and Foxp3^+^ CD4^+^CD25^+^ T cells (Treg cells) were monitored by flow cytometry at indicated time points. **(A)** Efficacy of Treg cell depletion. Plots were day 3 data following injection of anti-CD25 antibody, Foxp3^+^ CD4^+^CD25^+^ T cells were detected from peripheral blood (PBL), spleen (SPL), and draining lymph nodes (dLN). Results shown are representative of three experiments performed. **(B)** The distribution of Foxp3^+^ CD4^+^CD25^+^ T cells in naive mice. The percentages of Foxp3^+^ CD4^+^CD25^+^ T cells were detected from peripheral blood (PBL), spleen (SPL), and draining lymph nodes (dLN). Data are shown as mean ± SE. **(C)** Indicated time points show the kinetics of gradual replenishment of Foxp3^+^CD4^+^CD25^+^ T cells in PBL, SPL, and dLN following anti-CD25 injection. Data are shown as mean ± SD. Results are representative of two experiments performed.

*Foxp3*, described as a critical gene for Treg cells development and suppression function, is the most widely accepted signature of Treg cells (20, 21). We examined the distribution of Treg cells (CD4^+^CD25^+^Foxp3^+^ cell) in adult BALB/c mice in different compartments such as peripheral blood (PBL), spleen (SPL), and drain lymph nodes (dLN) in adult mice (Figures 1A,B). Thirty days later, following anti-CD25 treatment, Treg cells were gradually replenished in all three compartments including PB, SP, and dLNs. Within 3 months following injection of anti-CD25 antibody, Tregs cells in all three studied compartments recovered completely (Figure 1C). We did not see any autoimmune-like symptoms exhibited in anti-CD25 antibody injected mice such as weight loss, skin rashes, and increased hair loss.

### Pretreatment with anti-CD25 antibody during priming phase yields significantly enhanced immune responses to plasmid gp120 DNA

**Figure 2:**
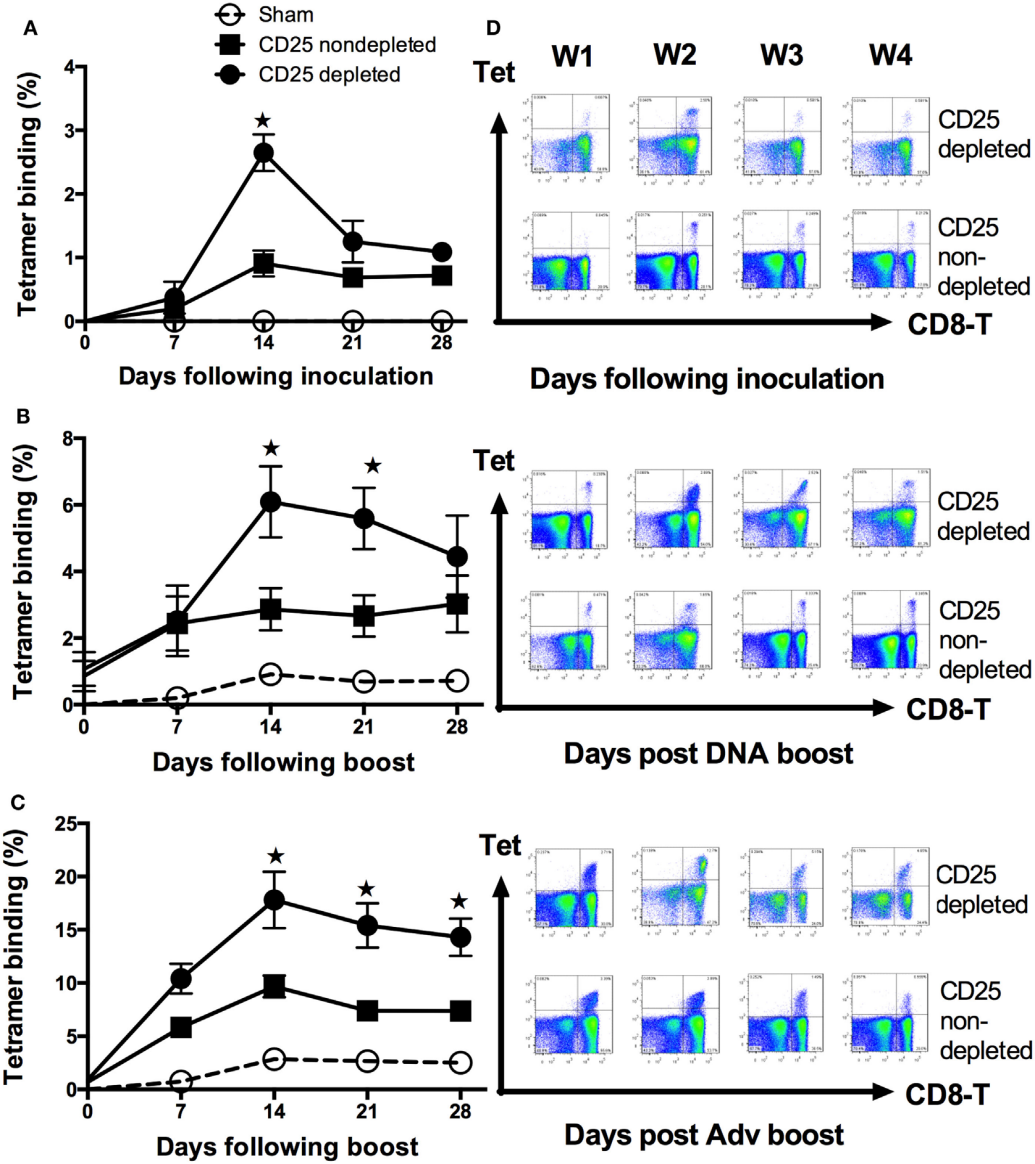
**Enhancement of antigen-specific CD8^+^ T-cell responses following depletion of natural Foxp3^+^CD4^+^CD25^+^ Treg cells in HIV-IIIB gp120 DNA immunization**. Kinetics of H-2D^d/^p18 tetramer staining of peripheral blood CD8^+^ T cells was monitored following depletion of natural FoxP3^+^CD4^+^CD25^+^ Treg cells. Data shown are representative of three experiments performed. Sham was used as an empty DNA with the same background as the DNA encoded HIVIIIB gene (refer to Materials and Methods). **(A)** Groups of mice (*n* = 4 mice/group) were first pretreated with either anti-CD25 mAb or control antibody in day 3 and in day 0 intramuscularly injected with 50 μg plasmid gp120 DNA vaccine construct. Tetramer for H-2D^d^/P18-specific CD8^+^ T-cell binding was then monitored at indicated time points (by weekly). The Treg cells depleted group had a threefold higher tetramer binding level in primary than the one in the control group at days 14 following plasmid gp120 inoculation (*p* < 0.05; Student’s *t*-test). **(B)** On day 42, mice (*n* = 4/group) previously immunized with gp120 DNA were boosted with homologous plasmid gp120 DNA. **(C)** Heterologous rAdv gp140 vaccine-elicited cellular immune responses were measured by tetramer binding to CD8^+^ T lymphocytes. Data are shown as mean ± SE. Arrows indicate boost immunizations. **(D)** Representative flow plots display percentages of CD8^+^tetramer^+^ T cells in the PBMCs between CD25-depleted and non-depleted groups at each time point of the injected mice. Plots are shown for the primary response (upper panel), homologous boost (middle panel), and the heterologous boost response (lower panel).

Animals were injected with the gp120 DNA following administration of anti-CD25 Ab or control antibody, the percentages of H-2D^d^/p18 tetramer (p18 tetramer) binding in CD8^+^ T cells from PBMCs were determined by weekly following DNA inoculation. At day 14 following DNA vaccine inoculation, anti-CD25 treated animals had a significantly higher percentage of p18 tetramer binding in CD8^+^ T cells (*p* < 0.05, Student’s *t*-test) (Figures 2A–D). No responses were observed in mice that received anti-CD25 antibody alone (data not shown).

### Immune response of homologous prime-boost vaccine is augmented by treatment with anti-CD25 antibody prior to priming vaccination

To examine the effect of anti-CD25 antibody administration, strategy of homologous prime-boost vector-based vaccines was employed. Three days prior to vaccinations, groups of BALB/c mice were injected with anti-CD25 Ab or controls by i.p., and then received plasmid gp120, i.m. At 6 weeks after priming, mice were boosted with the same plasmid gp120 that was used for priming, and p18 tetramer-specific CD8^+^ T cells from PBMCs were analyzed weekly after boosting. We demonstrated that, at the indicated time points (weeks 2 and 3 following boost), mice receiving anti-CD25 had elevated tetramer binding response (Figures 2B–D) (*p* < 0.05, Student’s *t*-test).

### Mice pretreated with anti-CD25 antibody prior to priming vaccination augments heterologous prime-boost vaccine immunogenicity

To assess the magnitude of immune response to gp120 DNA vaccine during heterologous prime-boost vaccine strategy in anti-CD25 Ab pretreated animals, mice were boosted with recombined adenovirus gp140 DNA (rAd5 gp140), 6 weeks later following priming with gp120 DNA construct. Tetramer binding in CD8^+^ T cells was statistically higher at all time points after day 14 (*p* < 0.05, Student’s *t*-test) in CD25-depleted mice (Figures 2C,D).

### Increase in the percentage of CD62L expression in P18 peptide-specific CD8^+^ T cells after treg cells depletion in mice

**Figure 3:**
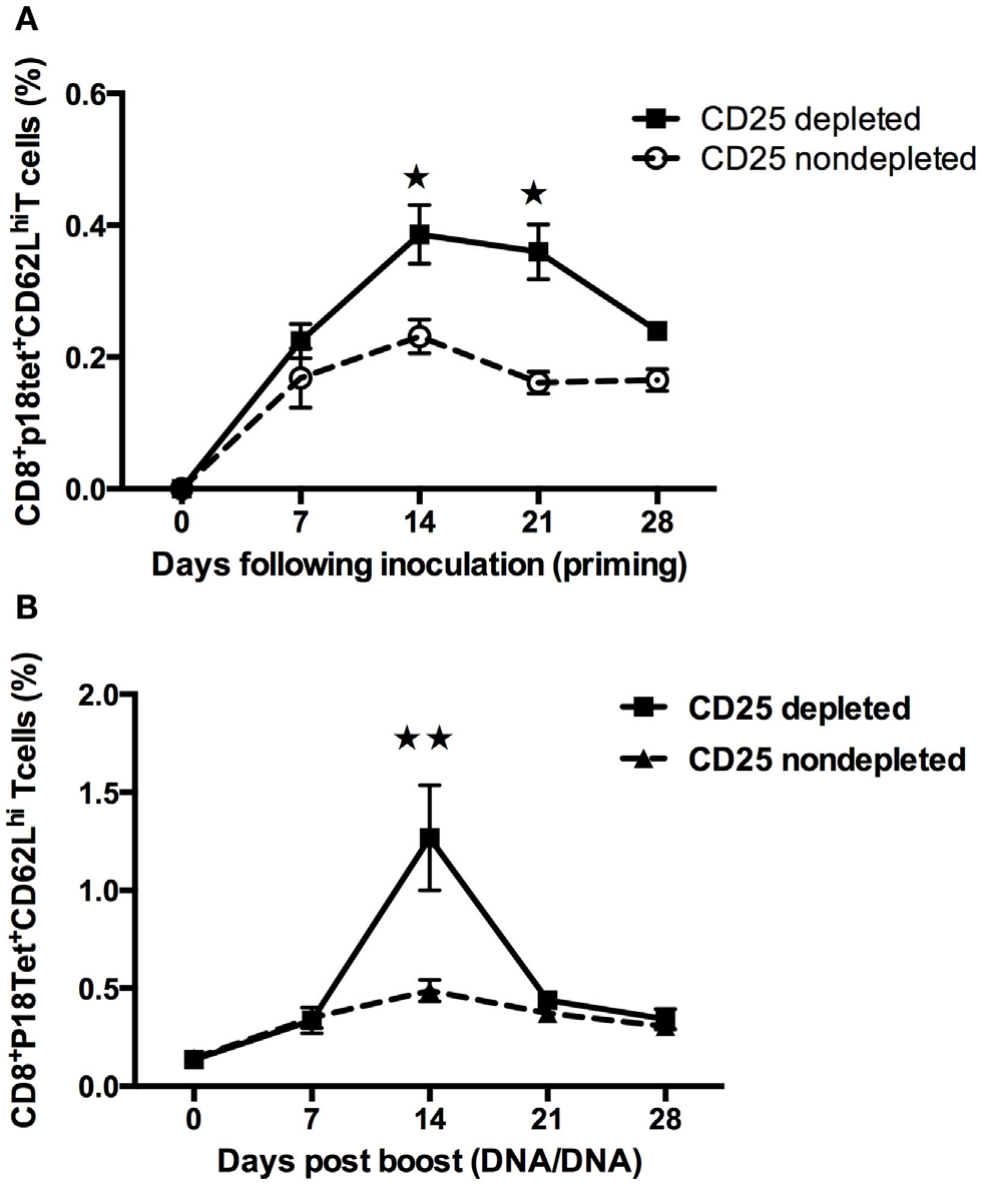
**Increases the percentage of CD62L expression in P18 peptide-specific CD8^+^ T cells after Treg cells depletion in mice**. Multiple lymph nodes including inguinal, mesenteric, and brachial obtained from groups of mice (*n* = 4 mice/group). **(A)** At day 0, Treg cells were depleted by administration of anti-CD25 antibody, and at day 3, plasmid gp120 DNA was injected. CD62L-specific tetramer-positive CD8^+^ T cells were measured. Differences in frequencies of tetramer in CD8 lymphocytes were statistically higher between 14 and 21 days (Student’s *t*-test with a cutoff of *p* = 0.05). **(B)** Mice at day 42 following gp120 DNA priming, then homologous gp120 DNA was boosted, the percentage of the gp120 antigen-specific H-2D^d^/p18^+^CD62L^+^CD8^+^ T cells frequencies was monitored weekly, at 14 days following inoculation of DNA vaccine reached a maximum and was statistically higher in CD25 injected group (*p* < 0.01, Student’s *t*-test). Data are expressed as the mean percentage ± SE. Results shown are representative of three experiments performed.

CD62L is one of the most prominently cited markers of recent antigen stimulating and homing for T-cell trafficking *in vivo*, which is also a marker for memory response development (22). To explore the expression of this molecule, kinetics of antigen-specific CD8^+^ T cells in PBMCs from multiple lymph nodes, including inguinal, mesenteric, and brachial, was studied. Following inoculation of the plasmid gp120 DNA, CD62L^+^H-2D^d^/p18 tet^+^ CD8^+^T cells in CD25-depleted mice reached a maximum at 14 days (*p* < 0.01, Student’s *t*-test) and declined significantly thereafter after homologous boost (Figure 3B). Compared to the boost phase, the pattern in the priming phase was slightly different: CD62L^+^H-2D^d^/p18 tet^+^ CD8^+^ T cells in CD25-depleted mice was significantly higher than in non-depleted mice at days 14 and 21 following plasmid DNA vaccine immunization (*p* < 0.05, Student’s *t*-test) (Figure 3A).

### Treg cells depletion leads to improved CD4^+^ T-cell response to gp120 vaccine

**Figure 4:**
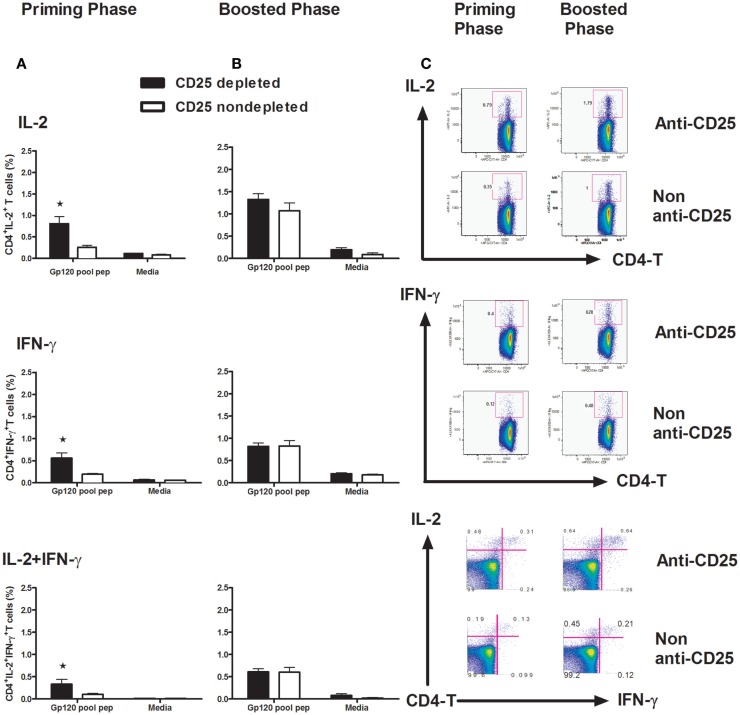
**Intracellular staining for IL-2 and IFN-γ in CD4^+^ T cells**. Groups of mice (*n* = 4 mice/group) Treg cells depleted at day 3 and non-depleted, plasmid gp120 DNA was injected **(A)** or **(B)** boosted on day 42 with homologous gp120 DNA. On day 14 following priming or post boost, mice were sacrificed and splenocytes were harvested, isolated, and exposed for 6 h to a pool of 47 overlapping peptides spanning the HIV-IIIB gp120 protein (2 μg/ml) or to media alone. The frequencies of intracellular IFN-γ^+^ CD4^+^ T cells, IL-2^+^CD4^+^ T cells, and IFN-γ^+^ IL-2^+^ polyfunctional CD4^+^ T cells were measured by an LSRII Flow machine, all cytokines show higher frequencies (*p* < 0.05) in CD25 depleted during primary phase, but not in boosted phase as determined by one-way ANOVA test. **(C)** Representative flow plots are shown with percentages in IL-2^+^ (upper panel), IFN-γ-positive (middle panel), and IL-2/IFN-γ double-positive populations in CD4 T lymphocytes. Numbers indicate the percentages of these cell populations and representative of the means of four mice per group ± SE. Results shown are representative of three experiments performed.

CD4^+^ T cells play a prominent role in enabling CD8^+^ T-cell activation, proliferation, and differentiation. They are also critical for CD8^+^ T-cell memory formation and maintenance. By producing IL-2, CD4^+^ T cells are thought to be necessary for programming the differentiation of fully functional CD8^+^ T-cell memory (5, 6, 23). In this study, we addressed the question of how Treg cells affect CD4^+^ T-cell function by detecting IL-2 and IFN-γ intracellularly. We harvested splenocytes at day 14 post vaccine inoculation, following a 6-h exposure to a pool of peptides spanning the gp120 protein. A one-way ANOVA showed the differences between the CD25-depleted and non-depleted animals. Significantly higher levels of IL-2, IFN-γ, and the poly IL-2^+^/IFN-γ^+^-CD4^+^ T cells were recorded in the priming phase in Treg cell-depleted mice (Figures 4A,C) *(p* < 0.05, one-way ANOVA). No difference for these cytokine expressions was demonstrated in memory phase (Figure 4B) (*p* > 0.05, one-way ANOVA). These data suggest that early CD4^+^ T-cell function is of critical importance for the establishment of an effective CD8^+^ T-cell response (24).

### Functional CD8^+^ T-cell immune response to gp120 DNA immunogen is improved in Treg cells depleted mice

**Figure 5:**
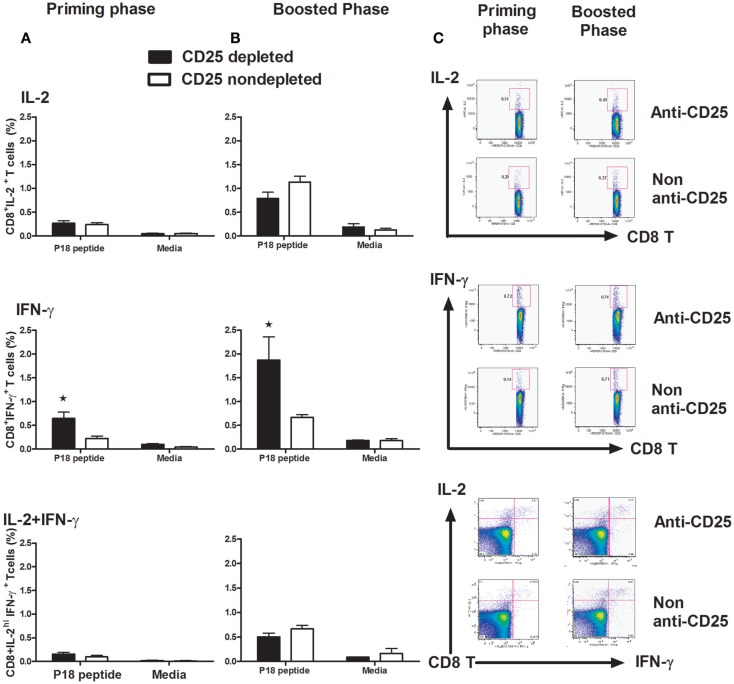
**Intracellular staining for IL-2 and IFN-γ in CD8^+^ T cells**. Groups of mice (*n* = 4 mice/group) with Treg cells depleted or non-depleted were first primed with plasmid gp120 DNA **(A)** or **(B)** boosted on day 42 with homologous gp120 DNA. On day 14 following priming or post boost, mice were sacrificed and splenocytes were harvested, isolated, and exposed for 6 h to the p18 peptides (2 μg/ml) or media alone. The percentage of intracellular IFN-γ^+^ CD8^+^ T cells, IL-2^+^CD8^+^ T cells, and IFN-γ^+^ IL-2^+^ dual function CD8^+^T cells was measured on a LSRII flow machine, we only see a statistically higher IFN-γ populations in both primary and memory phases (*p* < 0.05, one-way ANOVA test). Numbers indicate the percentages of these cell populations and representative of the means ± SE. **(C)** Representative flow plots are shown with percentages of IL-2-positive (upper panel), IFN-γ-positive (middle panel), and IL-2/IFN-γ double-positive populations in CD8 lymphocytes. Results shown are representative of three experiments performed.

To determine whether the depletion of Treg cells had an influence on CD8-T cell function, we harvested splenocytes at day 14 post vaccine administration both in the priming and boost phases. We observed that the cell population producing IFN-γ in CD8^+^ T cells stimulated by the p18 peptide was significantly increased in Treg cell-depleted mice in both priming and memory phases (*p* < 0.05, one-way ANOVA) (Figures 5A–C). However, no statistically significant differences were detected in IL-2 and IL-2/IFN-γ polyfunctional cell populations detected, neither in the priming phase nor in the memory phase (Figures 5A–C).

### Depletion of Treg cells shortens the duration of DNA antigen expression

**Figure 6:**
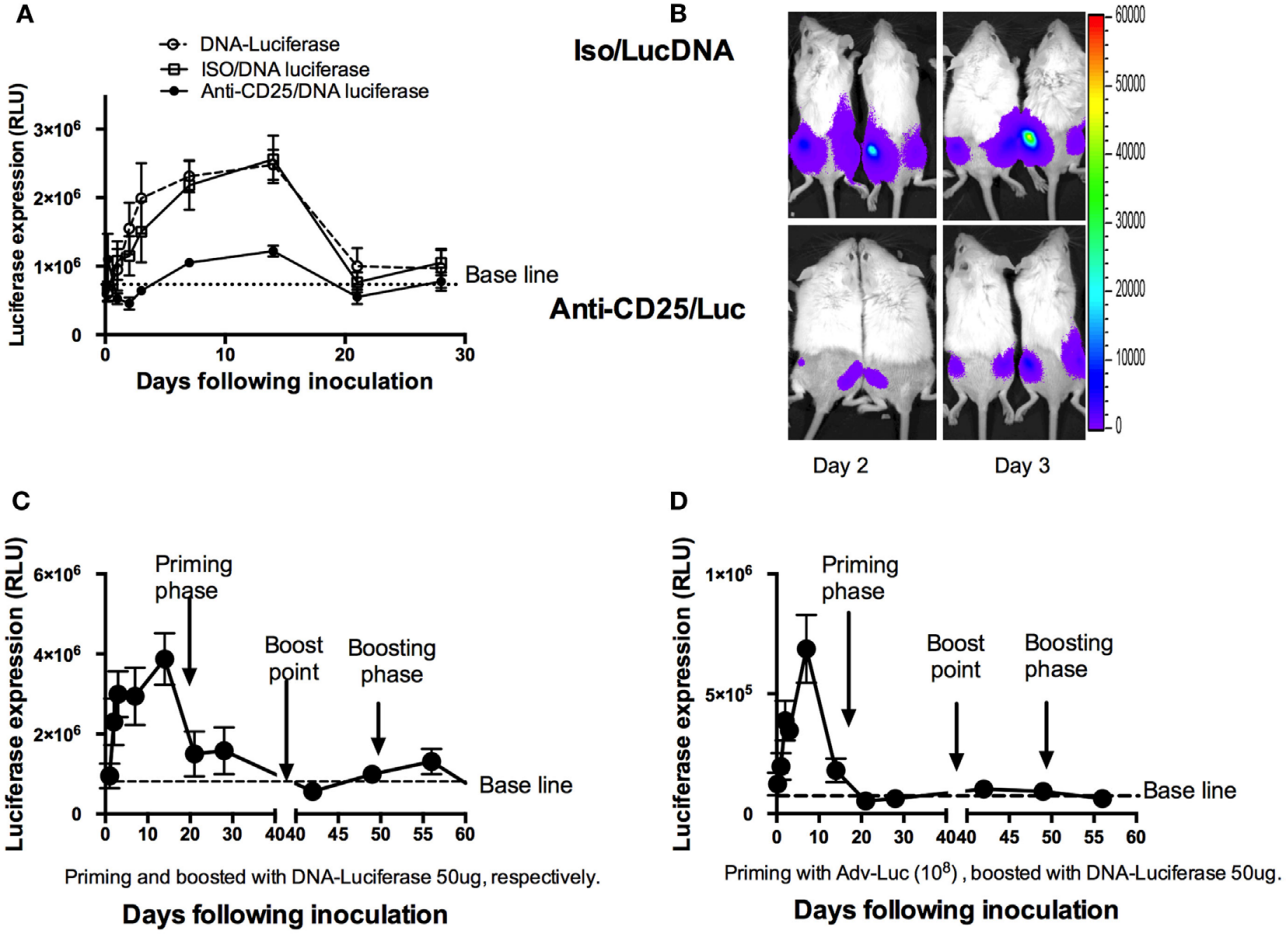
**Shortening DNA antigen duration by depletion of natural Treg cells prior to DNA luciferase immunization**. (**A)** At day 3, mice (*n* = 4 mice/group) were injected intraperitoneally with 1 mg of PC61 anti-CD25 monoclonal antibody, control antibody, or left as empty control group. At day 0, 50 μg plasmid luciferase was injected, and luciferase expressions were monitored by IVIS over the ensuing 28 days and were expressed as mean ± SD. Luciferase expression was significantly decreased from day 1 to day 14 during the ensuing 28 days’ observations in the anti-CD25 antibody injected group (*p* < 0.05, Student’s *t*-test). (**B)** Representative images of mice at day 2 and day 3, respectively, for luciferase expressions between CD25 depleted and non-depleted mice. **(C)** Mice (*n* = 4) were injected 50 μg DNA luciferase at both priming and recall phases (homologous boosted at day 42 following priming with DNA luciferase), respectively. IVIS monitoring at indicated time points. Dramatically reduced luciferase expression was determined at memory phase (less than 10 days). **(D)** Mice (*n* = 4) were injected 10^8^ adenovirus-encoded luciferase particles in priming phase; 42 days later, 50 μg DNA Luciferase (heterologous boost) was injected. Luciferase expressions were monitored by IVIS at indicated time points. The mean relative light unit (RLU) values expressed by groups of four mice ± SD.

Previous report has shown that responses in CD4^+^ T cells rather than CD8^+^ T cells were more dependent on antigens in proliferation (25). For CD8^+^ T cells, the development of long-lived memory with protective capacity was all programmed within the first 1–2 days of acute infection (7, 10, 26). To explore whether DNA antigen expression may also be affected by Treg cells, we first depleted Treg cells, and then injected mice with a plasmid that expressed luciferase. Luciferase expression was monitored by IVIS over the ensuing 28 days (Figure 6A), and the average luciferase expression was evaluated. To our surprise, luciferase expression reduced more drastically in the Treg cell-depleted mice (Figures 6A,B). Significantly decreased antigen expression was seen as early as day 1 with an extension to day 14 (*p* < 0.05, Student’s *t*-test). We then compared this expression pattern to regular memory phases including homologous (Figure 6C) and heterologous (Figure 6D) luciferase boost strategies. For this study, two groups of mice were first primed with DNA luciferase (DNA-Luc) and Adv encoded luciferase by i.m., respectively. Luciferase expressions were monitored at different time points as indicated in Figure 6 (Figures 6C,D). After 6 weeks (42 days), these two groups of mice were respectively boosted to inject with 50 μg DNA-Luc, i.m., the expressions of luciferase were continuously to be monitored following the ensuing observations. Surprisingly, in homologous boosted mice (Figure 6C), luciferase expressions were only detected in a very short time (less than 10 days) and showed with lower expression values compared to the priming phase. Almost no luciferase expressions were detected in heterologous boosted group (Figure 6D). This shortened antigen expression pattern in the memory phases was very similar to the one in a single Treg cell-depleted mice model in priming phase (Figure 6A).

### Treg cell does not affect CD8^+^ T-cell memory response following exposure to gp120 over 5 days

**Figure 7:**
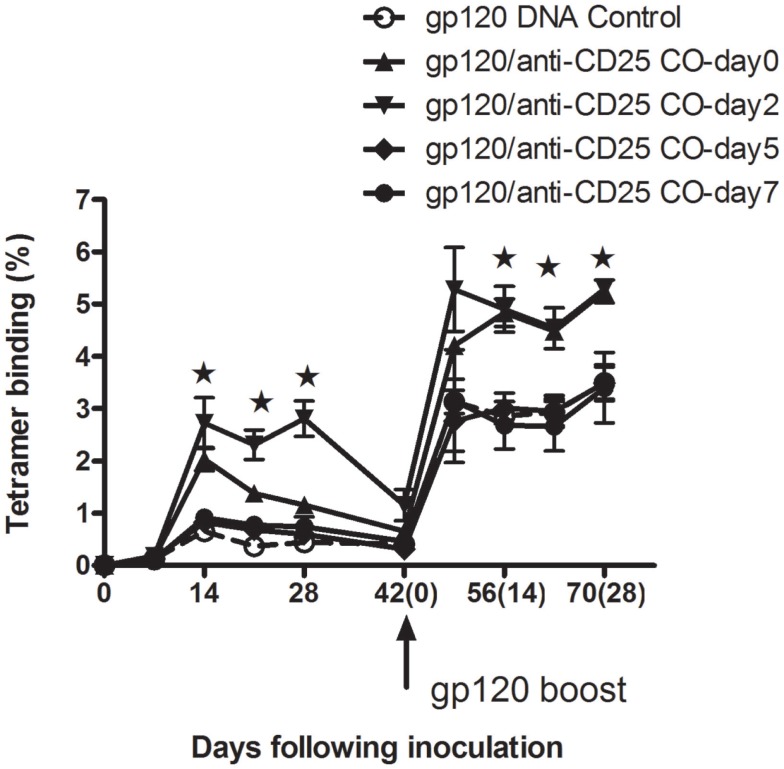
**Tregs depletion following mice exposure to plasmid gp120 at different time points**. Groups of mice (*n* = 4 mice/group) were injected with plasmid gp120 first. Then at day 0, 2, 5, and 7 following inoculation, mice were injected with anti-CD25 neutralizing antibody, respectively. CD8 T-cell responses were measured weekly by P18 tetramer bindings. Data are shown at day 0 and day 2 in anti-CD25-treated groups, CD8 T-cell memory responses were enhanced after boost 14 days (*p* < 0.05, Student’s *t*-test), while no memory responses boosted were seen for day-5 and day-7 groups. Results shown are representative of two experiments performed. Data are expressed as the mean percentages of tetramer-positive CD8^+^ T cells ± SE.

Having seen that Treg cells depletion prior to antigen exposure could enhance CD8^+^ T cells in both priming and memory phases, we were prompted to investigate whether Treg cells would affect immunogenicity following antigen exposures. Experiments were then designed to assess the tetramer binding ability in CD8 T cells. Five groups of mice were all immunized with gp120 DNA vaccine at day 0 first. With the exception of one group left as the DNA vaccine control, the four remaining groups of mice then respectively received anti-CD25 Abs at days 0, 2, 5, and 7 following plasmid gp120 immunizations. We found that, in groups of day 0 (gp120 and anti-CD25 antibody coinjected) and day 2 (mice received anti-CD25 Abs 2 days following DNA vaccine injection), CD8^+^ T-cell responses were all augmented both in priming and memory phases at indicated time points (Figure 7) (*p* < 0.05, Student’s *t*-test). Dunnett’s multiple comparison tests with a cutoff of *p* = 0.05 showed immune responses to be significantly higher in animals in the day 2 group during the primary phase. For day 5 and day 7 groups, no boosted effects were seen in animals (Figure 7), suggesting that Treg cells may have earlier effects on regulation of CD8^+^ T-cell immune responses.

## Discussion

The development of an effective vaccine against HIV has proved to be difficult, owing to our limited knowledge of the protective immunity needed to contract the infection. In order to develop vaccines against diseases such as AIDS, malaria, and tuberculosis, it is crucial to understand and to optimize the development of CTL responses to vaccines. More specific models such as genetically engineered Foxp3-DTR mice have been employed by several laboratories to study the specific role of Treg cells in primary and memory T-cell responses (18, 19). The major concern for depletion of Treg cells is the induction of autoimmunity. However, recent reports have shown some contradictions, by blockade or transient depletion of CD25 using the FDA-approved anti-CD25 antibody (Daclizumab): researchers do see an enhancement of immunity (27) but without induction of autoimmunity in cancer patients (27, 28). Nevertheless, more recent studies have also shown promising results by targeting the interaction between CCR4 expressed on Tregs cells and its ligands, CCL22 and CCL17, to inhibit transiently the recruitment of Tregs at the site of immunization. Importantly, this activity was observed in diverse models without noticeable side effects of autoimmunity. As CCR4 is also expressed on a variety of immune cells, including DCs and B cells, they may also deplete these kinds of cells (for details please see a review written by Bayry et al.) (29). Therefore, further study of the function of Treg cells including the mechanisms will merit its potential applications by avoiding potential feedbacks such as autoimmunity.

Regulatory T cells participate in the immune response to many and/or all infectious agents and immunogens. Although Treg cells can respond to a large variety of self-antigens, these responses may decrease the efficacy of protective immunity. Accumulating evidence indicates that immunity to viral infections such as HSV, HIV, and HCV are partially controlled by natural Treg cells including the efficacy of vaccines (30, 31). Depletion of Treg cells has been shown to enhance CD4^+^ T cells (11, 15), CD8^+^ T cells (26, 30–33), or both CD4^+^ and CD8^+^ T-cell responses (12, 13, 34). Likewise, Treg cells have also been reported to control the intensity and magnitude of secondary responses to infectious pathogens such as *Listeria*, *Leishmania*, and HSV (31–33). In the present study, we have demonstrated that, in the absence of Treg cells, enhanced immune responses were paralleled with increased antigen-specific CD62L^+^H-2D^d^/p18 tet^+^ CD8^+^ T-cell population during both primary and memory phases. This is well in agreement with the notions that the cells survived contraction for seeding the memory pool (memory precursor) and may have already been presenting at the peak of expansion (Figure 3) (35, 36). This population may contribute greatly to the enlargement of the antigen-specific memory pool (Figure 3) (4, 35). A more recent study by Buchholz et al. (37) suggested that families showing greater expansion contained more effector and effector memory T cells, whereas small families were predominantly composed of central memory T cells. This study has also indicated that the fate of single naive CD8^+^ T cells is determined at an early stage by either random internal variation between individual naive T cells or by random (external) variation in the environmental cues that they receive (37). Our present study is very well in line with their findings. We have shown in the absence of Treg cells enhanced CD8 T-cell responses (Figure 2A), and CD4 T cells at an early time (i.e., the priming phase) produce larger number of IL-2 and IFN-γ (Figure 4A), which might be useful in helping to establishing robust immune responses during memory stimulations.

A number of mechanisms by which Treg cells contribute to the suppression of immune response have been extensively studied. One of the most notable consequences of the modulation of immune response is to enhance pathogen survival and a long-term persistence (4). Recent studies have confirmed and added to the concept that a relatively short encounter with antigen may launch CD8^+^ T cells into a program of proliferation and differentiation without continuous antigenic stimulation, under the assumption that the original signal could have already programmed the long-term fate of the subsequent CTL population (38, 39). Consistent with this concept, we have shown that, in the absence of Treg cells, the DNA expression was disrupted from day 1 following the injection of plasmid luciferase during the ensuing observation period (Figure 6A). Interestingly, previous studies on T-cell memory response have drawn the conclusions that functional antigen-dependent CD8^+^ T-cell memory populations formed after acute infections, while chronic infections led to generation of inefficient or poor quality of CD8^+^ T-cell memory (40, 41). In fact, through the observation of DNA expression, we found for the first time that a shortened antigen duration occurred in the absence of Treg cells (Figure 6A), with this *in vivo* DNA-Luc expression displaying a pattern similar that of the regular memory response (Figures 6C,D). One important implication of this result is that it better explains why depletion of Treg cells is able to enhance immune response during pathogen invasions and immunogen vaccinations.

A number of mechanisms have been shown to limit the expression of vaccine vectors *in vivo*. We have previously shown that type I interferons can dampen DNA-Luc expression under the treatment of plasmid GM-CSF. In that study, the DNA-Luc used is exactly the same as in the present study (1). It is still unclear whether or not type I interferons can be one of the causative reasons to shorten the duration of the plasmid luciferase expression, as we did not check the type I interferon-production level following the depletion of Treg cells. Although immune-mediated destruction of Ag-producing muscle fibers has been reported following immunization with plasmid DNA expressing a surface-expressed hepatitis B virus Ag (HBsAg), this destruction was not seen with a plasmid DNA expressing the intracellular protein luciferase (18). Herein, we have excluded the possibility that shortened antigen expression with such a rapid speed and short time is due to the specific clonal expanded CD8 T cells. Rather, our data are consistent with a mechanism by which the function of CD4 T cells influences the duration of depot antigen and the *in vivo* clearance of plasmid DNA (42). This study has also demonstrated that the control of DNA antigen expression can result in accelerated contraction, differentiation, and greater memory CD8 T-cell responses as well (42). Additionally, data from a previous study showed that Fas-mediated apoptosis limited *in vivo* vaccine antigen expression (19). The reasons why the luciferase antigen disappears more rapidly under anti-CD25 treatment are still largely unknown. Further study will merit the elucidation of the mechanisms underlying antigen duration-associated immune responses. Indeed, in this study, in the absence of Treg cells, we have demonstrated a strong correlation of enhancement of CD8^+^ T-cell responses with shortened DNA antigen duration in DNA vaccine in both priming and secondary phases, which also provided strong evidence to support the notion in memory T-cell development. In other words, depletion of Treg cells during priming phase, enhanced immune response is likely adding one more set of memory responses to the immune system.

Moreover, this notion is further supported by results of early-elevated intracellular cytokine profiles in CD4 T cells. As CD4^+^ T cells can play an essential role in response to primary antigen challenges for initially expanding CD8^+^ T cells (43), programming CD8^+^ T-cell differentiation into long-lived protective memory (44, 45). Consistent with this notion, our present work has shown that, by depletion of Treg cells (Figure 4), increased IFN-γ and IL-2 producing CD4^+^ T-cell populations only appeared in primary immunization. The results suggested that, early in the process of immune responses, these cytokines may play an important role in helping memory CD8 T-cell formation. The expansion function of IFN-γ in Ag-specific T-cell populations has been extensively studied (46–49). As for IL-2, the essential factor for Treg cell survival, which has also been shown to be indispensable to program the differentiation into functional CD8^+^ T-cell memory at early time (50–52).

Despite the fact that numerous studies have been shown to enhance immune responses by depleting Treg cells, and although the anti-CD25 antibody has been approved used for therapeutic applications, the mechanisms underlying the adjuvant effects of anti-CD25 neutralizing antibody are still largely unknown. Herein, we are for the first time displaying that, by administration of anti-CD25 antibody, the pattern of DNA vaccine-induced immune response is similar to the one in a regular memory phase, which better explains why the depletion of Treg cells is able to enhance immune response during pathogen invasions and immunogen vaccinations.

Taken together, our findings support the conclusions that Treg cells control DNA vaccine immunogenicity at an early time via antigen duration and functional CD4^+^ T-cell responses. Depletion of Treg cells during priming phase-enhanced immune response is likely adding one more set of memory response to the immune system.

## Materials and Methods

### Animals and immunizations

Six- to eight-week-old female Balb/c mice were obtained from The Jackson Laboratory (Bar Harbor, ME, USA) and Charles River Laboratories (Wilmington, MA, USA). Mice were maintained under pathogen-free conditions, and experimental protocols were approved by the Harvard Institutional Animal Care and Use Committee. For experiments conducted in China, all protocols were approved by the ethics committee of Shandong Academy of Medical Sciences (Jinan, China) and performed in compliance with the EC regulations (O.J. of E.C.L358 12/18/1986) and the NIH standards (Guide for the Care and Use of Laboratory Animals, National Institutes of Health’s publication 86-23, revised 1996). For plasmid DNA immunizations, 50 μg of pVRC-HIV-1 Env IIIB gp120, the complete CXCR4-tropic HIV-1 HXB2 Env IIIB (GenBank accession no. K03455), was cloned into the pVRC vector as previously described (42). Plasmid DNA was prepared using an endotoxin-free Qiagen Giga-prep kit (Qiagen, Valencia, CA, USA). rAdvgp140 and rAdv-luciferase were provided by Dr. Barouch. For immunizations, 50 mg of plasmid DNA in 100 ml of sterile saline was divided between quadriceps muscles by intramuscular (i.m.) inoculation. The plasmid DNA-Luc construct was prepared as previously described (53). This vector contains the GL4.10 luciferase gene (Promega, Madison, WI, USA) or the empty pVRC plasmid (NIH Vaccine Research Center, Bethesda, MD, USA) were injected in 100 μl of sterile saline divided between the left and right quadriceps muscles (54).

### Antibodies and reagents

PC61 (anti-CD25) was purchased from Bioexpress (NH) for *in vivo* administration. Antibodies purchased from BD Pharmingen were PerCP anti-CD8α (clone; 53-6.7), Alexa Flour 700 anti-IFN-γ (clone; XMG1.2), APC anti-IL-2 (Clone; JHS6-5H4), and PE-Cy7 anti-CD4 (clone; GK1.5). Antibodies purchased from eBioscience were FITC anti-CD4 (RM4-5), APC anti-CD25 (eBio,7D4), and *PE anti-Foxp3 FJK-16s*.

### Tetramer staining assay

Tetrameric H-2D^d^ complexes folded around the HIV-1 IIIB V3 loop P18 epitope peptide (P18-I10 or RGPGRAFVTI) (1, 55) were prepared and used to stain P18-specific CD8^+^ T cells as previously described (53). Mouse blood was collected in RPMI 1640 containing 40 U/ml heparin. Following lysis of RBCs, 0.1 μg PE-labeled D^d^/P18 tetramer in conjunction with APC-labeled anti-mouse CD8 mAb (Ly-2, Caltag, San Francisco, CA, USA) was used to stain P18-specific CD8^+^ T cells. The cells were washed in PBS containing 2% FBS and fixed in 0.5 ml PBS containing 1.5% paraformaldehyde. Samples were analyzed by LSRII flow machine (BD Biosciences, CA, USA) and gated CD8^+^ T cells were examined for staining with the H-2D^d^/P18 tetramer.

### FoxP3 T cells intracellular staining

Staining procedures followed the product instructions from eBioscience. Briefly, cells isolated from PBL, SPL and lymph node (about 1 million PBMC) were first subject to staining surface molecular for CD4 and CD25 for 20 min and then fixed and permeated using Cytofix/Cytoperm buffer (BD biosciences). Finally, cells were stained with PE-labeled, anti-mouse Foxp3 antibody and analyzed on an LSRII flow machine (BD Biosciences).

### Splenocyte stimulation and intracellular cytokine staining

Splenocytes harvested from individual mice and red blood cells were lysed by using ACK lysing buffer. The cells were then washed with PBS + 2% FBS, counted, and stained with PE-conjugated H-2D^d^/p18 tetramer. The cells were then resuspended (4 × 10^6^ cells per tube) in RPMI 1640 medium (Cellgro, Herndon, VA, USA) supplemented with 10% FBS, 25 mM HEPES, 2 mM l-glutamine, 20 U of penicillin per milliliter, 20 μg of streptomycin per milliliter, 1 mM sodium pyruvate, and 0.1 mM non-essential amino acids. For CD8^+^ T-cell stimulation, cells were incubated with Golgi Plug (2 μl/ml), anti-CD28 (2 μg/ml), anti-CD49d (2 μg/ml), and p18 peptide (2 μg/ml). For CD4^+^ T-cell stimulation, instead of the p18 peptide, the cells were incubated with 2 μg of Env peptide pool per milliliter. The pool consisted of 47 overlapping 15-mer peptides spanning the HIV-1 IIIB gp120 protein (Centralized Facility for AIDS Reagents, Potters Bar, UK), and each peptide had a concentration of 2 μg/ml. Unstimulated cells were incubated with all the above reagents except for the peptides. As a positive control, splenocytes were incubated with PMA (2 μg/ml), ionomycin (10 μg/ml), and Golgi Plug. The cells were incubated at 37°C for 6 h and then washed with PBS + 2% FBS, stained with PE-conjugated H-2D^d^/p18 tetramer for 15 min, followed by addition of antibodies specific for cell surface molecules for an additional 15 min. Permeabilization was performed overnight with Cytofix/Cytoperm solution (BD Biosciences). Cells were washed with 1× Perm/Wash buffer (BD Biosciences) and then stained with anti-cytokine mAb. After an additional washing step with 1× Perm/Wash buffer, the cells were fixed in 2% formaldehyde-PBS. Samples were collected on an LSR II instrument (BD Biosciences) and analyzed using FlowJo software (Tree Star).

### *In vivo* bioluminescence measurement

Animals were injected i.p. with 100 μl of a 30 mg/ml solution of firefly luciferin in PBS (Xenogen, Alameda, CA, USA), 100 μl of a 20 mg/ml ketamine, and a 1.72 μg/ml xylazine mixture (1). After 20 min, imaging was performed using the *In vivo* Imaging System (IVIS) Series 100 (Xenogen) with an integration time of 1 min. Luminescence measurements were made using Living Image software (Version 2.50.1, Xenogen).

### Depletion of Treg cells in different times following exposure to antigen immunization

Following gp120 DNA vaccine immunization at day 0, 2, 5, and 7, 6- to 8-week-old female Balb/c mice were divided into four groups (*n* = 4) and i.p. injected 1 ml (concentration, 1.0 mg/ml) anti-CD25 neutralizing antibody to observe the splenocytes priming and memory CD8^+^ T-cell responses by detecting CD8^+^ T cells for P18 tetramer binding.

## Statistics

The statistical significances of differences between groups were determined by Student’s *t*-test, one-way ANOVA, or Dunnett’s multiple comparison tests using the GraphPad Prism program (version 6.03). A value of *p* < 0.05 was considered statistically significant. Error bars represent the standard error or standard deviation of the mean as described in the figure legends.

## Author Contributions

LQ designed and performed experiments and drafted the manuscript. GJ and JH analyzed part of the results, and NLL agreed to publish the data.

## Conflict of Interest Statement

The authors declare that the research was conducted in the absence of any commercial or financial relationships that could be construed as a potential conflict of interest.
